# Active Methamphetamine Use is Associated with Transmitted Drug Resis-tance to Non-Nucleoside Reverse Transcriptase Inhibitors in Individuals with HIV Infection of Unknown Duration

**DOI:** 10.2174/1874613600701010005

**Published:** 2007-10-22

**Authors:** Edward R Cachay, Niousha Moini, Sergei L Kosakovsky Pond, Rick Pesano, Yolanda S Lie, Heidi Aiem, David M Butler, Scott Letendre, Wm. Christopher Mathews, Davey M Smith

**Affiliations:** 1 University of California, San Diego, La Jolla, California, USA; 2 Monogram Biosciences, Inc., South San Francisco, California, USA; 3 Veterans Administration San Diego Healthcare System, San Diego, California, USA

**Keywords:** HIV, NNRTI, transmitted drug resistance, methamphetamine.

## Abstract

**Background:**

Frequent methamphetamine use among recently HIV infected individuals is associated with transmitted drug resistance (TDR) to non-nucleoside reverse transcriptase inhibitors (NNRTI); however, the reversion time of TDR to drug susceptible HIV may exceed 3 years. We assessed whether recreational substance use is associated with detectable TDR among individuals newly diagnosed with HIV infection of unknown duration.

**Design:**

Cross-sectional analysis.

**Methods:**

Subjects were enrolled at the University California, San Diego Early Intervention Program. Demographic, clinical and substance use data were collected using structured interviews. Genotypic resistance testing was performed using GeneSeq™, Monogram Biosciences. We analyzed the association between substance use and TDR using bivariate analyses and the corresponding transmission networks using phylogenetic models.

**Results:**

Between April 2004 and July 2006, 115 individuals with genotype data were enrolled. The prevalence of alcohol, marijuana and methamphetamine use were 98%, 71% and 64% respectively. Only active methamphetamine use in the 30 days prior to HIV diagnosis was independently associated with TDR to NNRTI (OR: 6.6; p=0.002).

**Conclusion:**

Despite not knowing the duration of their HIV infection, individuals reporting active methamphetamine use in the 30 days prior to HIV diagnosis are at an increased risk of having HIV strains that are resistant to NNRTI.

## BACKGROUND

Twenty-five million people worldwide illicitly used amphetamines in 2005, and rates of abuse in North America are increasing [1]. Methamphetamine use is associated with increased confidence and sexual activity [2, 3], and it is the most widely used recreational drug among men who have sex with men (MSM) in California, USA [4]. Methamphetamine use has been associated with a doubling of the risk of HIV acquisition [5], higher blood viral loads, alterations in antiretroviral (ARV) medication concentrations, and greater high-risk sexual behaviors, which may lead to HIV superinfection [6-8].

HIV transmitted drug resistance (TDR) reduces the number of effective ARV medications available for an HIV infected individual before ever considering ARV therapy [9, 10]. In the United States, reported rates of TDR to at least one class of ARV medications ranges from 8 to 24% [9-3]. In California, the majority of HIV TDR results in decreased susceptibility to the non-nucleoside reverse transcriptase inhibitor (NNRTI) class of ARV medications [12, 14].

Among MSM, there is a confluence of high prevalence of HIV infection, TDR and methamphetamine use [12, 15]. Indeed, frequent methamphetamine use among recently HIV infected MSM is associated with TDR to NNRTI [16].

Most individuals who are newly diagnosed with HIV infection do not know how long they have been infected. As only a minority of HIV infected people are diagnosed during acute infection, there is often a variable period of time from acquisition until diagnosis. This delay in diagnosis could theoretically decrease detection of TDR due to reversion of the virus to a susceptible genotype; however, the time required for reversion to occur is often long (>3 years) [10], especially in the male genital tract [17]. Individuals with HIV TDR and a long delay in diagnosis may forward propagate their TDR virus. We therefore conducted the present study to test the hypothesis that active methamphetamine users who are newly diagnosed with an HIV infection of unknown duration and ARV naive would have higher rates of HIV TDR than their non-methamphetamine using counterparts. We also sought to characterize the association between active methamphetamine use and HIV TDR as one of correlation or causation by investigating HIV transmission networks with regards to TDR and methamphetamine use.

##  METHODS

###  Study Participants

Participants were recruited between April 2004 and July 2006 at the University of California, San Diego Early Intervention Program. The study was approved by the Human Research Protection program at University of California, San Diego. Participants were ARV naïve adults in whom HIV was diagnosed within the three months prior to enrollment to our cohort. The dates of HIV infection were unknown in this clinical cohort. Assessments were conducted in the first month following enrollment. All participants were tested for sexually transmitted infections (STI) including urethral gonorrhea and Chlamydia (LCx STD system, Abbott Laboratories, Abbott Park, Illinois, USA), syphilis (rapid plasma reagin), and Hepatitis C virus (ORTHO® HCV 3.0 ELISA). Additionally, blood HIV (Amplicor, Roche) and CD4 counts (flow cytometry) were measured. Risk factors for exposure to HIV and demographic characteristics were collected by structured interview for all study participants.

###  Drug Resistance Testing

Resistance testing was performed and interpreted with population-based sequencing of *pol* (Geneseq^Tm^ Monogram Biosciences). Drug resistance was identified according to Genseq^Tm ^resistance algorithm (Monogram Biosciences) and the International AIDS Society 2006 guidelines [18]_._Transmitted drug resistance was defined as the presence of any major resistance mutation associated with any of the following ARV classes: nucleoside reverse transcriptase inhibitors (NRTI), NNRTI or protease inhibitors (PI).

### Substance Use

Participants were asked about substance use by a health educator in a structured clinical evaluation, as part of routine medical intake interview. The format of the questions emphasized comprehensibility, response burden and acceptability of language. The questions were asked in either English or Spanish dependent on the language most often used by the participant. Individuals were asked specifically about the their preferred types and routes of recreational drug use, including alcohol, marijuana, methamphetamine, cocaine,ecstasy (MDMA -3,4-methylenedioxy-N-methylamphetamine), opiate, and GHB (gamma hydroxybutyrate) use. Substance use was categorized as “never”, “ever used” or “active substance use in the 30 days prior to the time of HIV diagnosis”. Individuals were also queried about using any ARV medications either before or after any high risk sexual exposures in order to prevent HIV infection.

### Phylogenetic Analysis

We sought molecular evidence for TDR and clustering among participants reporting active substance use. The *pol *sequence alignment (entire coding region of protease and the first 305 codons of reverse transcriptase) was screened for evidence of phylogenetic discordance among sequence fragments using a maximum likelihood method [19]. Because the sequences in this study showed strong (p<0.001, Kishino-Hasegawa test) evidence for at least one breakpoint (nucleotide 479), our subsequent analyses were based on methods that do not assume a single phylogenetic tree. We estimated synonymous genetic distances between all pairs of sequences by fitting a codon-substitution model to each pair with maximum likelihood, allowing us to correct for the possibility of convergent evolution in response to similar selective pressures. We applied four F_st_ tests [20, 21] to assess whether sequences were genetically segregated based on an attribute such as substance use or drug resistance. All sequence analyses were performed with the HyPhy software package (http: //www.hyphy.org).

### Statistical Analysis

We examined bivariate relationships between substance exposure, potential confounding factors, and drug resistance in contingency table analysis. Analyses were performed using STATA version 9.1. To avoid over-fitting, multivariate analyses were not pursued given the results of our bivariate analysis.

## RESULTS

During the study period, 168 eligible subjects were enrolled at the University of California, San Diego Early Intervention Program, however genotypic resistance data were available in only 115 participants. One-hundred and twelve participants were men and 3 were women. Fifty percent of patients were non-white. The main risk factor for HIV acquisition was being MSM (89%). Median age was 32 years. Demographic characteristics of our cohort were similar to those of the individuals in San Diego County who are newly diagnosed with HIV [22]. The median blood viral load and CD4 cell count of study participants were 4.50 log_10_ copies/ml (range: 1.70 to 5.88) and 422 cells/mm^3 ^(range: 55 to 1404).

The three most common substances “ever used” for the study participants were alcohol, marijuana and methamphetamine (98%, 71% and 64%, respectively). Of these, alcohol was the most commonly reported substance actively being used within the 30 days prior to HIV diagnosis (69%), followed by methamphetamine (24%). Six of twenty-eight participants reporting methamphetamine use in the prior 30 days admitted using it intravenously. Three people were current users of psychotropic drugs at the time of HIV diagnosis, but none of them reported methamphetamine use. Similarly, no participant reported ever taking ARV medications prior to HIV diagnosis.

The prevalence of TDR to at least one class of ARV medication was 23% (n=26). Resistance to NNRTI (n=13, 11%) or NRTI (n=12, 10%) was relatively common, while resistance to PI was less so (n=3, 2.6%). No major PI mutations were found, but three individuals had more than 5 minor PI mutations detected and were classified as PI resistant [18]. Only two participants had HIV showing genotypic resistance to both NNRTIs and NRTIs. The most common resistance-associated mutation was K103N, appearing in 7 of 13 (54%) participants with NNRTI resistance.

In bivariate analyses, frequent methamphetamine use (within 30 days prior to HIV diagnosis) was marginally associated with TDR to any class of HIV drugs (OR: 2.4, p= 0.055). Despite similar frequency of TDR for both NRTI and NNRTI in the cohort, frequent methamphetaime use was strongly associated only to TDR of NNRTI (OR= 6.6, 95%, CI 1.9 – 22.2; p=0.002). None of the other covariates was associated (p>0.10) with TDR, so we did not proceed with multi-predictor modeling (Table **1**).

### Sequence Analysis Results

On average, a pair of *pol* sequences from our cohort were 4.2% (SD=0.9%, range 0.1-7.2%) divergent, as measured by synonymous substitutions per 100 nucleotides. We defined putative transmission clusters as groups of sequences that were 1% or less divergent from at least one other sequence in the cluster. Three such clusters (10, 7 and 2 sequences) were identified. In the larger clusters 7 of 10 and 5 of 7 sequences were from methamphetamine users, although these proportions were not significantly different from the overall prevalence of methamphetamine use in the entire sample (p>0.35, Fisher’s exact test). For all sequences sampled in the population, there was no significant association between genetic relatedness and substance use, whether considered wholly or severally, nor between genetic relatedness and genotypic resistance to any of the three classes of drugs.

## DISCUSSION

This study found that in our cohort of newly diagnosed, ARV naïve individuals of unknown duration of infection, those who reported using methamphetamine within the 30 days prior to their HIV diagnosis were more likely to have TDR to NNRTI than those who did not. Methamphetamine users do not represent a homogenous group of individuals, in our cohort, those who reported using methamphetamine within 30 days of their HIV diagnosis most likely represented active or on-going users, while those who reported using “methamphetamine ever” but not within 30 days of their diagnosis most likely represented sporadic users. These two groups differ in both the psychosocial reasons for methamphetamine use as well as in the type and frequency of high risk sexual behaviors [2, 23]. More frequent methamphetamine use has been associated with higher rates of unprotected sexual intercourse, trading sex for money or drugs, and having partners of unknown or serodiscordant HIV status [24-26]. But these are risk factors for HIV acquisition, not for TDR [27].

Our phylogenetic analyses did not yield evidence of transmission linkage of TDR among methamphetamine users; however, our sample was probably too small to draw firm conclusions through these molecular epidemiology techniques. Despite the lack of statistically significant phylogenetic linkages, the positive association between active methamphetamine use and NNRTI TDR is provocative.

The acquisition of a resistant virus by the recipient partner suggests certain characteristics about the source partner, such as HIV positive status, access to medical care, ARV exposure, and perhaps medication adherence [24, 28, 29]. Also, active methamphetamine use by recipient partners is most likely associated with methamphetamine use by the source partners [24, 26]. Taken together, we theorize that active methamphetamine use is associated with acquisition of HIV TDR at multiple levels, as proposed in Fig. (**1**) [4, 5, 8, 30-36]. Possibly secondary to cognitive and behavioral changes induced by methamphetamine use or side effects induced by select NNRTI, the source partner may had reduced ARV compliance, leading to evolution of drug resistant mutations [30, 31]. The lower genetic barrier to resistance to currently available NNRTI, can occur at higher levels of adherence than for the development of resistance to PI [31, 32]. In particular, Efavirenz can produce a state of long restless sleep, vivid dreams, and nightmares that individuals try to avoid after an episode of methamphetamine use [33]. Additionally, NNRTI resistance in the source partner may be more likely to be transmitted to the recipient partner than other forms of ARV class resistance because NNRTI resistance-associated mutations have less impact on viral replication capacity [32, 34]. For example, the common NRTI resistance-associated mutations, M184V and T215F/Y, typically impair HIV replication capacity in the absence of ART [35,36]; therefore, these mutations may be more likely to revert to drug susceptible forms [37, 38] than NNRTI resistance-associated mutations, and may allow these NNRTI mutations to persist for longer after discontinuation of NNRTI selective pressure, increasing the risk of NNRTI TDR [17, 39]. This is supported by our data where most individuals in our cohort showing NNRTI TDR did not also show NRTI TDR, even though a source partner with NNRTI resistance probably would have also developed NRTI resistance, as these ARV’s are often used together in clinical practice [9, 10].

Not knowing the duration of infection in our cohort could be a methodological limitation because any reversion of a drug-resistance mutation to wild-type would cause an underestimation of the prevalence of TDR, and this may occur more frequently in other drug classes [12, 37]. However, not knowing the exact duration of HIV infection is often the case in most clinical scenarios, and without knowing the duration of infection in our cohort, we still found a rate of HIV TDR comparable to that seen with primary HIV infection [9, 10, 13], which is entirely consistent with the long duration of TDR, particularly with NNRTI [12, 37].

Our findings must be tempered by the following limitations. First, we have no direct information in the source partner. Second, our sample size may not have been sufficiently large to detect associations between methamphetamine use and PI or NRTI TDR. Indeed, based on the observed proportions of PI and NRTI among individuals unexposed to methamphetamine within 30 days prior to HIV diagnosis, our study only had 33% and 70% power to detect an association between metamphetamine exposure and PI and NRTI TDR when the odds ratio was greater than or equal to 3. Although subtle associations could have been missed, a previous report with 300 patients also did not find any association between methamphetamine use and PI or NRTI TDR [16]. Third, as with all location specific cohort studies, our results cannot be generalized to all individuals with HIV infection because of potentially unique aspects of the location of our cohort [12].

Despite these limitations, this study has substantial clinical implications. It reinforces the need for routine resistance testing among individuals newly diagnosed with HIV, particularly recent methamphetamine users, even when the duration of infection is unknown. Furthermore, it questions the clinical practice of prescribing NNRTI-based regimens to individuals with active methamphetamine use. It follows that if the source of the TDR is the source partner and if the recipient partner is actively using methamphetamine so is the source partner [24, 26], then perhaps PI-based regimens instead of NNRTI should be used to treat HIV infection among individuals who continue to use methamphetamine so as to limit the spread of NNRTI TDR.

## Figures and Tables

**Fig. (1):**
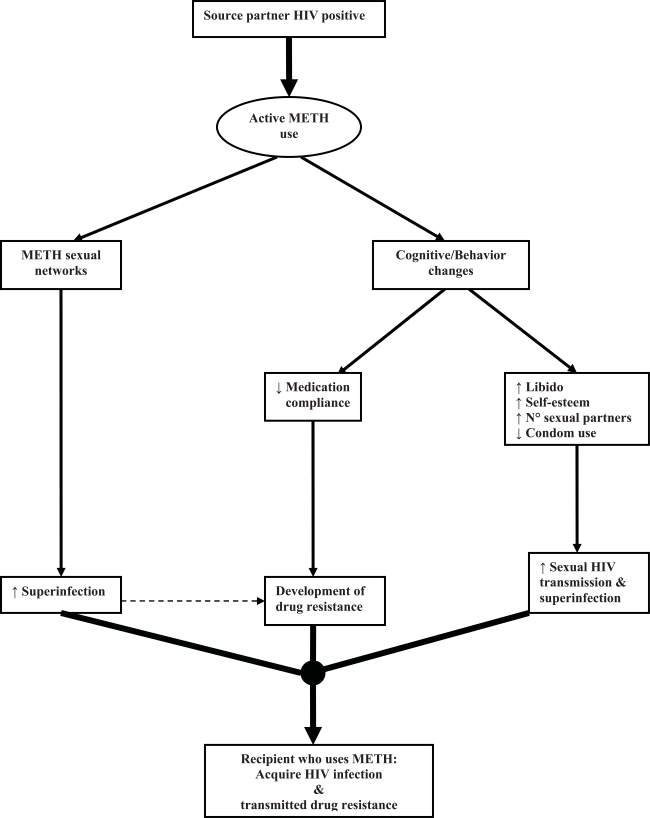
Theoretical model of casual association between active methamphetamine use and transmitted drug resistance (METH = Methamphetamine; ↑ = Increase; ↓= Decrease).

**Table 1:** Unadjusted Effects of Baseline Charactersitics on Risk of NNRTI Mutation at Enrollemnt (N = 115 Treatment Naïve Pa-tients)

Characteristic	Odds ratio	95% Confidence Interval	P	Category P
Age (years)				0.84
20-25	1	0.19 - 4.68	0.94	
26-31	0.93	0.18 - 4.35	0.87	
32-37	0.87	0.07 - 3.08	0.43	
≥ 38	0.47			
Race/Ethnicity				0.17
Non-Hispanic white	1	0.85 - 10.83	0.09	
Hispanic	3.03	0.1 - 9.15	0.96	
Other/Unknown	0.95			
Risk factor				0.23
MSM ^a^	1	0.84 - 17.23	0.08	
IDU^b^	3.8	0.38 - 10.90	0.41	
MSW^c^ not IDU	2.03			
CD4 (cells/mm^3^)				0.35
55-310	1	0.61 - 51.60	0.13	
311-421	5.63	0.45 - 41.31	0.2	
422-611	4.32	0.30 - 31.90	0.34	
≥ 612	3.12			
HIV RNA(log 10 copies/mL)				0.34
1.70 - 3.72	1	0.23 - 9.73	0.67	
3.73 - 4.49	1.5	0.62 - 18.49	0.16	
4.50 - 4.94	3.4	0.13 - 7.65	0.97	
≥ 4.95	0.96			
Substance use^d^				0.09
Methamphetamine ever	3.4	0.72 - 16.18	0.12	
**Methamphetamine_30^e^**	6.6	**1.94 - 22.21**	**0.003**	**0.002**
Sexual Transmitted Diseases^f^				0.87
Syphilis	1.14	0.23 - 5.71	0.87	
Hepatitis C	1.3	0.15 - 12.04	0.8	0.8

^a^MSM = Men who have sex with Men; ^b^IDU = Intravenous drug user; ^c^MSW = Men who have sex with women; ^d^Other substances included in the univariate analysis but yield no significant associations were: Alcohol, Marijuana, Cocaine, Gamma hydroxybutyrate, Ecstasy, Opiates. Estimates were in comparison to the “never” reference category; ^e^Reported active methamphetamine use within 30 days of HIV diagnosis; ^f^There were no subjects diagnosed with urethral Chlamydia or gonorrhea infections In Bold substance use that has a significant association with transmitted drug resistance.
